# TERT promoter mutations in melanoma render TERT expression dependent on MAPK pathway activation

**DOI:** 10.18632/oncotarget.10634

**Published:** 2016-07-16

**Authors:** Andrelou F. Vallarelli, P. Sivaramakrishna Rachakonda, Jocelyne André, Barbara Heidenreich, Laurence Riffaud, Armand Bensussan, Rajiv Kumar, Nicolas Dumaz

**Affiliations:** ^1^ INSERM, U976, Skin Research Centre, Hôpital Saint-Louis, Paris, F-75010, France; ^2^ Université Paris Diderot, Sorbonne Paris Cité, UMRS976, Paris, F-75010, France; ^3^ Department of Internal Medicine, School of Medicine Sciences, State University of Campinas (UNICAMP), 13083-970, Campinas, SP, Brazil; ^4^ Division of Molecular Genetic Epidemiology, German Cancer Research Center, 69120 Heidelberg, Germany

**Keywords:** melanoma, telomerase, ETS1, MAPK pathway, BRAF

## Abstract

The mechanism of telomerase re-activation in cancer had remained elusive until the discovery of frequent mutations in the promoter of the TERT gene that encodes the catalytic reverse transcriptase subunit of telomerase. We investigated the regulation of TERT expression in melanoma cell lines and our results show that promoter mutations render TERT expression dependent on MAPK activation due to oncogenic BRAF or NRAS mutations. Mutations in the TERT promoter create binding sites for ETS transcription factors. ETS1, expressed in melanoma cell lines, undergoes activating phosphorylation by ERK at Thr38 residue as a consequence of constitutively activated MAPK pathway. We demonstrate that ETS1 binds on the mutated TERT promoter leading to the re-expression of the gene. The inhibition of ETS1 resulted in reduced TERT expression. We provide evidence that the TERT promoter mutations provide a direct link between TERT expression and MAPK pathway activation due to BRAF or NRAS mutations via the transcription factor ETS1.

## INTRODUCTION

Melanoma arises from the malignant transformation of melanocytes that involves numerous genetic alterations affecting multiple signaling pathways including MAPK (Mitogen Activated Protein Kinase), PI3K (Phosphoinositide 3-kinase), cAMP and cyclin D1/CDK4 [1]. The MAPK pathway plays a major role in melanoma proliferation and survival and is activated in the majority of melanoma tumors through mutations in BRAF and to some extent in NRAS. Mutations in BRAF constitute an early event and occur in over 80% of benign nevi; however, the sustained BRAF or NRAS expression in human melanocytes leads to oncogene-induced senescence [2, 3]. A critical step in the process of cellular immortalization remains the reactivation of telomerase.

**Figure 1:**
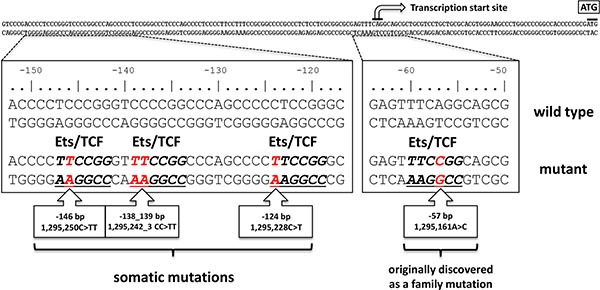
Distribution of mutations in the TERT promoter The sporadic and germline mutations found in melanoma are shown in red below the TERT core promoter. The Ets/TCF consensus motifs created by mutations are underlined. The numbering above the sequence relates to the start codon (ATG) of TERT whereas the standardized positions of mutations on chromosome 5 are indicated in the boxes. The transcription start is indicated by an arrow.

Telomerase, a ribonucleoprotein complex consisting of a catalytic subunit, reverse transcriptase (TERT) and an RNA component (TERC) acts canonically through maintenance of telomere homeostasis and chromosomal integrity [4]. TERT expression is tightly regulated, present during early embryonic development but remains repressed in most adult human somatic cells. However, over 90% of human cancers present reactivation of telomerase [5]. While benign and dysplastic nevi contain little or no telomerase, the majority of melanoma display substantial telomerase activity [6]. The mechanism for the cancer-specific reactivation of telomerase has remained unclear. The discovery of activating somatic mutations within the core promoter region of the TERT gene has provided an insight into the possible cause of telomerase re-expression in some cancer types. The initial TERT promoter mutation discovery came from a causal A > C germ line mutation at –57 bp (from ATG start site; Chr 5:1,295,161 hg19 co-ordinate) in a large melanoma family. The TERT promoter mutations were also found to occur as somatic alterations at a high frequency in melanoma tumors from unrelated patients [7, 8]. Subsequent studies showed occurrence of somatic TERT promoter mutations in a wide range of cancer types [9]. In melanoma, those somatic mutations have been associated with increased TERT expression, increased Breslow thickness, tumor ulceration, and poor disease-free and melanoma-specific survival [10, 11]. Another study suggested that TERT promoter mutations might constitute early secondary alterations [12]. The recurrent mutually exclusive C > T somatic mutations in the TERT promoter at –124 (1,295,228) bp and –146 (1,295,250) bp, like familial mutation, result in the creation of a binding motif for the ETS (E26 transformation-specific) transcription factors with consequent tumor-specific increased TERT expression (Figure 1). The ETS family of transcription factors contains 28 members, which can be divided into 13 subfamilies of one to three members. All proteins of the family share a conserved DNA binding domain that mediates monomeric binding to the ETS binding consensus sequence, consisting of an invariant GGA(A/T) core and, often, an extended sequence CCGGAA(A/T) [13, 14]. At least nine of the 28 ETS family proteins can be phosphorylated by ERK, and in eight of those cases that modification leads to increased transcriptional activation [15]. In melanoma the TERT promoter mutations occur together with BRAF and NRAS mutations more frequently than per chance, suggesting a link between TERT expression and MAPK pathway activation during immortalization of melanocytes via ETS proteins [10]. Several ETS members have been shown to bind to the sites created by mutations at the –124 and –146 positions *in vitro*. The transcription factors that have been shown to bind the sites include ETS1, ETS2, ELF1, ELF2, ETV6, p52 NF-κB and GABPA, however, no study has so far shown link between TERT expression and MAPK activation [16–18].

In this communication, we report that TERT expression is dependent of MAPK pathway activation only in melanoma cell lines carrying the TERT promoter mutations. Our data show expression of ETS1 and its phosphorylation in an ERK-dependent manner in all melanoma cell lines included in the study. We found that ETS1 binds to the TERT promoter with mutations but not with wild type sequence. ETS1 down-regulation partially inhibits TERT expression in cell lines with the TERT promoter mutations. Our study provides evidence of a direct link between TERT expression and MAPK pathway activation through the ETS1 transcription factor in melanoma cells carrying a TERT promoter mutation.

## RESULTS

**Figure 2:**
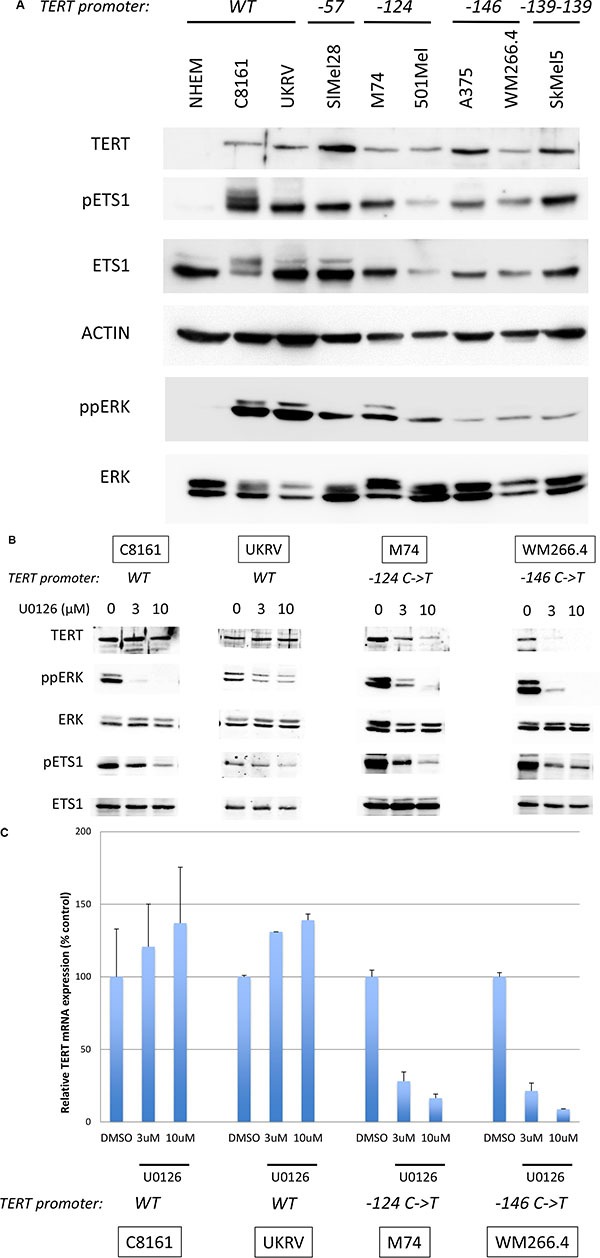
TERT expression is dependent on MAPK pathway activation (**A**) Levels of TERT, phosphorylated ETS1 (pETS1), total ETS1 (ETS1), phosphorylated ERK (ppERK), total ERK (ERK) and actin were analyzed by western blotting in melanocytes (NHEM) and melanoma cell lines. The TERT promoter mutation status is indicated above the blots. (**B**) Melanoma cell lines were treated for 48 hrs with 3 μM, 10 μM of U0126 or DMSO (0). Levels of TERT, phosphorylated ERK (ppERK), total ERK (ERK), phosphorylated ETS1 (pETS1), total ETS1 (ETS1) and actin were analyzed by western blotting. (**C**) Melanoma cell lines were treated for 24 hrs with 3 μM, 10 μM of U0126 or DMSO (0). TERT mRNA levels were quantified by real-time PCR normalized to GAPDH. Values are mean ± s.d. of two experiments assayed in duplicate. The TERT promoter status is indicated underneath the bar-charts.

To investigate a link between MAPK pathway activation and TERT expression in presence or absence of TERT promoter mutation, we performed experiments on nine melanoma cell lines with or without TERT, BRAF and NRAS mutations (Table 1). We first showed that normal human epidermal melanocytes (NHEM) did not express TERT; whereas, melanoma cell lines expressed different levels of TERT (Figure 2A). We observed no difference in the level of TERT protein in cells lines with and without promoter mutations. Both C8161 and UKRV-Mel21 cell lines also expressed TERT, despite the absence of promoter mutation. Similarly, there was no correlation between TERT expression level and heterozygosity or homozygosity of mutations at the –57, –124, –146 or –138_139 positions. Similar results were seen at the mRNA level with quantitative RT-PCR (data not shown). To evaluate the role of MAPK activation in TERT expression, four representative cell lines with (M74, WM266.4) or without (C8161, UKRV-Mel21) TERT promoter mutations were treated with the MEK inhibitor U0126. Western blots confirmed that U0126 inhibited ERK phosphorylation (Figure 2B). The effect of U0126 on TERT mRNA level and protein were analyzed using real-time RT-PCR and western blotting, respectively. ERK inhibition was associated with a reduced TERT protein expression in cell lines carrying a TERT promoter mutation (M74, WM266.4) but not in the C8161 and UKRV-Mel21 cell lines, which do not carry such mutation (Figure 2B). This decrease in TERT expression was also observed at mRNA level (Figure 2C) suggesting an inhibition at the transcriptional level. No inhibition of TERT mRNA was seen upon U0126 treatment in C8161 or UKRV-Mel21 cell lines, confirming that TERT expression, in the absence of TERT promoter mutations, is independent of MEK/ERK. Moreover, similar results were obtained using another MEK inhibitor, trametinib, and the BRAF inhibitor vemurafenib in melanoma cell lines with BRAF mutations (Supplementary Figure S1). The data suggest that, at least in the investigated melanoma cell lines, TERT expression is dependent on the activation of the MAPK pathway in the presence of TERT promoter mutations. To identify the link between MAPK pathway activation and TERT transcription, we investigated the expression of different transcription factors of the ETS family in melanoma cell lines. We found that ETS1, which has previously been shown to be involved in development and invasion of melanoma, was expressed in melanocytes and in all melanoma cell lines that were investigated (Figure 2A and data not shown). Furthermore, ETS1 was constitutively phosphorylated at threonine 38 in all melanoma cell lines. The phosphorylation at threonine, which activates ETS1, was dependent on ERK, shown by the activity of MEK inhibitor U0126 (Figure 2B). That result suggested that ETS1 could be the transcription factor linking the activation of the MAPK pathway to the expression of TERT in melanoma cell lines harboring TERT promoter mutations.

**Table 1:** Mutational status of cell lines used in this study

Cell Line	TERT promoter	BRAF	NRAS
NHEM	WT	WT	WT
501Mel	–124 C > T	V600E	WT
A375	–146 C > T	V600E	WT
C8161	WT	WT	Q61K
M74	–124 C > T	V600E	WT
Sk-Mel5	–138_139CC > TT	V600E	WT
Sk-Mel28	–57 A > C	V600E	WT
UKRV-Mel21	WT	WT	WT
WM266.4	–146 C > T	V600D	WT

**Figure 3:**
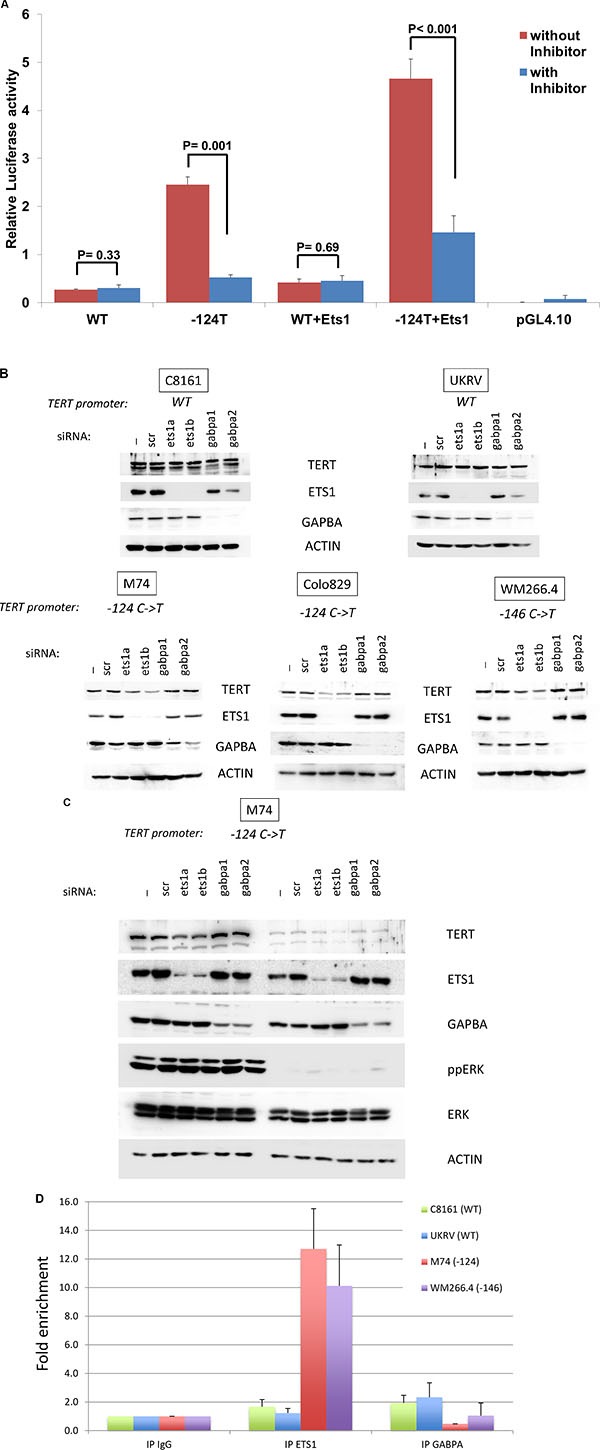
ETS1 binds to the TERT promoter and control its expression (**A**) Luciferase reporter constructs containing wild type TERT promoter sequence (WT) and sequence with mutant allele for the –124T mutant (-124T) were transfected in triplicate into UKRV-Mel21 cell line with and without plasmids with ETS1 sequence. To determine the effect of MEK inhibitors, in parallel experiments cells after 6 hours of transcfection were treated with Trametinib. Cells were harvested 64 hours post transfection and reporter expression was analyzed using the Dual-Luciferase assay system. The constructs without either reporter gene did not show any activity and were used as controls. (**B**) Melanoma cell lines were transfected without any siRNA (-), a siRNA control (scr), siRNA targetting ETS1 (ets1a and ets1b) or GABPA (gabpa1 and gabpa2). After 72 hrs, levels of TERT, ETS1 GABPA and ACTIN were analyzed by Western Blotting. The TERT promoter status is indicated above the blots. (**C**) M74 melanoma cell line was transfected as above. 24 hrs later cells were treated with DMSO or 3 μM of U0126. After 48 hrs of treatment, levels of TERT, ETS1 GABPA and ACTIN were analyzed by Western Blotting. The TERT promoter status is indicated above the blots. (**D**) Melanoma cell lines were formalin fixed and harvested. Chromatin precipitated by anti-ETS1, anti-GABPA or control IgG was reverse-cross-linked, and the obtained genomic fragments were quantified by real-time PCR. Values are presented as fold enrichment over the mean value of control IgG. Error bars represent standard deviations.

To confirm this hypothesis, we first determined the effect of ETS1 over-expression on promoter activity of the –124T mutant allele using luciferase reporter assays in the UKRV-Mel21 cell line. The first results from the assays showed that the reporter construct with the mutant allele showed higher promoter activity than the construct with wild type sequence (10-fold; *t*-test *P* 0.001); however, the addition of MEK inhibitor diminished the promoter activity in the construct with –124 C > T mutation (5- fold; *t*-test *P* 0.001; Figure 3A) and not in the construct without mutation. In a parallel experiment, the cells were co- transfected with ETS1 plasmid and reporter constructs with or without –124T promoter mutant allele. The results showed that ETS1 overexpression resulted in increased luciferase activity only with the reporter construct with the mutant allele, compared to the cells without mutant construct (11-fold; *t*-test *P* 0.002). Introduction of Trametinib, a MEK inhibitor, reversed the increase in promoter activity due the mutation in the presence of ETS1 overexpression (3.2-fold; *P* < 0.001). Increased expression of ETS1 in cells transfected with ETS1 plasmids was confirmed with real-time PCR (Supplementary Figure S2).

To evaluate the role of ETS1 on the endogenous TERT promoter, we used RNA interference to inhibit ETS1 expression in C8161, Colo829, M74, UKRV-Mel21 and WM266.4 cell lines. Two different siRNAs reduced ETS1 protein level by more than 70% in all cell lines. This reduction was associated with a 50% to 60% decrease in TERT expression in Colo 829, M74 and WM266.4 cell lines carrying a mutant TERT allele but not in C8161 and UKRV-Mel21, which harbored wild-type TERT promoter (Figure 3B and Supplementary Figure S3). These results suggest that ETS1 is, at least in part, responsible for TERT expression in melanoma cell lines with a mutant TERT promoter. As GABPA has been previously shown to drive efficient TERT transcription in cell lines with the mutations [16], we tested whether inhibition of GABPA by RNA interference decreased TERT expression. Although, two different siRNAs reduced GABPA protein level by more than 70% in all cell lines, TERT expression remained unaffected (Figure 3B and Supplementary Figure S3). To confirm the ERK-ETS1-TERT connection, we combined ETS1/GABPA depletion with MEK inhibition. U0126 inhibited ERK activation and reduced TERT expression, which was not further decreased by ETS1 or GABPA depletion (Figure 3C). To confirm that ETS1 is specifically recruited to the mutant TERT promoter, we performed ETS1 ChIP in M74 and WM266.4 cell lines both with TERT mutation, and C8161 and UKRV-Mel21 cell lines both without corresponding mutation. We showed that the TERT mutation induced a ten- to twelve-fold increase in ETS1 binding to the TERT promoter compared to the wild-type allele (Figure 3D). Using ChIP assays, we could only detect weak binding of GABPA on the TERT promoter and it was not associated with promoter mutation. As a control, we evaluated the binding of GABPA to RACGAP1 and KIF20A promoters, two promoters that have recently been shown to be direct targets of GABPA [19]. We showed by ChIP assays that both promoters bound GABPA in melanoma cell lines (Supplementary Figure S2) demonstrating the efficacy of the assays to detect GABPA binding. These data demonstrate that ETS1 is selectively recruited to the mutant TERT allele in melanoma cell lines and induce an allele-specific activation of TERT.

## DISCUSSION

Cellular immortalization is a multistep process and a major step in cancer development. It involves the sustained expression of telomerase, which gives cancer cells an infinite capability to divide through maintenance of telomeres. Although the reactivation of telomerase has a key role in the immortalization process, the mechanism underlying telomerase re-activation in cancers had remained elusive. However, the discovery of frequent mutations in the promoter of TERT highlighted a possible mechanism of catalytic subunit of reverse transcriptase expression in cancer cells. Functional studies have shown that the presence of TERT promoter mutations leads to failure of TERT repression upon cellular differentiation; in multiple cell lines, the mutant TERT promoter has been shown to cause massive epigenetic changes [20, 21].

However, the probable association between activation of TERT expression due to the promoter mutations and MAPK pathway activation has remain unexplored. Here, we show that the promoter mutations render the expression of TERT dependent on the activation the MAPK pathway. The MAPK pathway is activated mainly through oncogenic mutations of BRAF or NRAS, which are early events in melanoma development and in absence of other alterations lead to oncogene induced senescence. The acquisition of TERT promoter mutation in BRAF or NRAS mutated melanoma probably allows the re-expression of TERT leading to the immortalization process on path to melanoma development, together with other alterations such as inactivation of cyclin-dependent kinase inhibitor 2A (CDKN2A). TERT promoter mutations create novel binding sites for the ETS family of transcription factors. Several ETS proteins can be phosphorylated by ERK, and this modification activates their transcriptional activation [15]. We investigated the role of ETS1 transcription factor, which has previously been involved in the development and invasion of melanoma. ETS1 plays an important role in cancer progression due to its ability to activate the transcription of metastasis-, angiogenesis- and invasion-associated genes [22]. *ETS1* gene expression has been associated with tumor progression in various tumors such as thyroid, pancreas, liver, lung and breast carcinomas, and melanoma [23]. ETS1 is expressed in melanoblasts in normal adult melanocytes and in transformed cells; however, its role in melanoma progression is unclear. ETS1 has been reported either as a valuable diagnostic/prognostic marker [24] or as molecule with no clear association with clinical outcome [25]. In the present study, we showed that ETS1 is expressed in melanoma cell lines and is constitutively phosphorylated by ERK on Thr38 in melanoma cell lines due to the activation of the MAPK pathway associated with BRAF or NRAS mutations. Thr38 phosphorylation results in enhanced transactivation by preferential recruitment of the coactivators CREB binding protein (CBP) and p300 [26]. We demonstrated that ETS1 binds at the site created by the promoter mutations leading to TERT expression; the inhibition of ETS1 resulted in reduced TERT expression. These results are in accordance with recent data showing binding of ETS1 to TERT mutant promoter [17]. Amongst the several ETS1 members that have been shown to bind at the sites created by the mutation at –124 position, GABPA has been shown to drive efficient TERT transcription [16]. In this study, we could detect the expression of GABPA in melanoma cell lines; however, its expression did not decrease upon MEK inhibition (Figure 3C and data not shown). This observation is in conformity with a previous study that compared MAPK specificity across all ETS family proteins and shows that GABPA has very few MAPK interacting domains for phosphorylation by ERK, JNK and p38α kinases [27]. We also showed that inhibition of GABPA by RNA interference does not decrease TERT expression and we could only detect weak binding of GABPA on the TERT promoter by ChIP assays, which did not associate with the promoter mutation. The reason for the discrepancy between our results and a previous study remains unclear [16]. GABPA could be in a complex preventing binding of the antibody by masking the epitope, or epigenetic modifications of the TERT promoter in our cells could prevent binding of the transcription factor [21]. Nevertheless, our experiments with siRNA targeting GABPA suggest that, at least in the cell lines tested, GABPA does not stimulate TERT expression even in the presence of a mutation at the –124 or –146 positions. However, ETS1 inhibition induced only a partial reduction of TERT expression (Figure 3B) suggesting that other transcription factors may be involved in TERT expression in melanoma cells.

Our results have important implications for understanding the mechanism through which telomerase is reactivated in tumors cells. We provide evidence that TERT promoter mutations via ETS1 form a direct relationship between TERT expression and MAPK pathway activation through BRAF or NRAS mutations. This probably participates in cellular immortalization during melanoma development.

## MATERIALS AND METHODS

### Cell culture and transfection

Normal neonatal human epidermal melanocytes (NHEM; Cascade Biologics, Nottinghamshire, United Kingdom) were cultured in medium 154 supplemented with human melanocyte growth supplement (Cascade Biologics). Human melanoma cell lines were cultured in DMEM or RPMI 1640 medium (Gibco, ThermoFisher Scientific) supplemented with 10% FBS, penicillin (100 Units/ml)/streptomycin (100 μg/ml) antibiotics, and 2 mM L-glutamine. All melanoma cell lines were genotyped to verify their authenticity. siRNA were transfected using Lipofectamine RNAiMAX (Invitrogen) following the manufacturer instructions. Sequences of the siRNA are in Supplementary Table S1.

### Luciferase reporter gene constructs and expression vector

For reporter luciferase assays a 2.5 kbp region of TERT locus (chr5:1,294,815–1,297,313, hg19 coordinates) was amplified using genomic DNA. The amplified region included 2209 bp of promoter, followed by 219 bp of exon 1 and 60 bp of intron 1. The C > T mutation at –124 position was generated using Quik-change site-directed mutagenesis kit (Invitrogen) and cloned into pGL4.10 vector as described previously [28]. The ETS1 coding sequence in pENTR221 was obtained commercially and subcloned into pDEST26 Gateway expression vector.

### Luciferase reporter assay

For reporter assay, UKRV-Mel 21 cells were seeded in 12-well plates and transfected with Lipofectamine 2000 (Invitrogen), 500 ng of reporter construct (WT or –124 C > T) and 50 ng of pRL-actin in triplicates. The pRL expressing renilla luciferase was used as an internal control for normalization of luminescence values. Promoter-less vector (pGL4.10[luc2]) and non-transfected cells were used as negative controls. The three plasmids (WT, –124 C > T and promoter-less vector) were assayed separately with or without MAPK inhibitor (Trametinib) and additionally with or without Ets-1 overexpression. For Ets1 overexpression, the corresponding cells were cotransfected with 100 ng of PDEST26-ETS1 expression plasmid that expressed human Ets1 coding sequence (GenBank: AY893450). To determine the effect of MEK inhibitor, the cells were treated with Trametinib dissolved in DMSO at a final concentration of 1 μM, 6 hours after transfection. As a control, DMSO was used in other batch of cells that were not treated with Trametinib. Cells were harvested 30 hours post transfection using 1x passive lysis buffer (Promega) and reporter expression was analyzed using the Dual-Luciferase assay system (Promega). The relative ratio of firefly luminescence to renilla luminescence was calculated to normalize the variations across samples. Statistical differences were determined using two-sided *t*-test in R.

### Reverse transcription and real-time PCR

Total RNA was extracted from melanoma cell lines (RNeasy Mini Kit, QIAGEN) and treated with DNase (ThermoFisher Scientific); cDNA was prepared using the Themoscript kit (ThermoFisher Scientific). TERT mRNA level was quantified by real-time PCR using the Power SYBR Green kit (applied biosystems) normalized to GAPDH. Sequences of the primers are in Supplementary Table S1.

### Protein expression and antibodies

Cells were lysed in RIPA and the proteins were subjected to an SDS-PAGE and western blot analysis was carried out according to standard protocols using the following antibodies: TERT (Santa Cruz technology and Thermo Fisher Scientific), PhosphoERK (Sigma), ERK (Millipore), PhosphoETS1 (Sigma), ETS1 (Bethyl) and GABPA (Santa Cruz technology). Antibodies were visualized using the SuperSignal West Pico Chemiluminescent Substrate (ThermoFisher Scientific).

### ChIP

Cells were cultured in 10-cm plates to approximately 80% confluence, and fixed with 1% formaldehyde. Chromatin Immunoprecipitation was carried out using the Pierce Agarose ChIP Kit (Thermo Fisher Scientific) following the manufacturer instructions. The genomic fragments were quantified by real-time PCR using the Power SYBR Green kit (applied biosystems) and primers surrounding the –124 and –146 TERT promoter mutations (Supplementary Table S1). Primers for the RACGAP1 and KIF20A promoters have been described previously [19]. The antibodies used included, ETS1, GABPA (Santa Cruz technology) and rabbit IgG control (Abcam).

## SUPPLEMENTARY MATERIALS FIGURES AND TABLE


