# Supplementary material for: TERT promoter mutations in melanoma render TERT expression dependent on MAPK pathway activation
Source: Oncotarget. 2016 Jul 16;7(33):53127–36. doi: 10.18632/oncotarget.10634 (PMC5288173; doi:10.18632/oncotarget.10634)
Supplement: Supplementary file 1 [file oncotarget-07-53127-s001.pdf]

TERT promoter mutations in melanoma render TERT expression dependent on MAPK pathway activation

Supplementary Materials

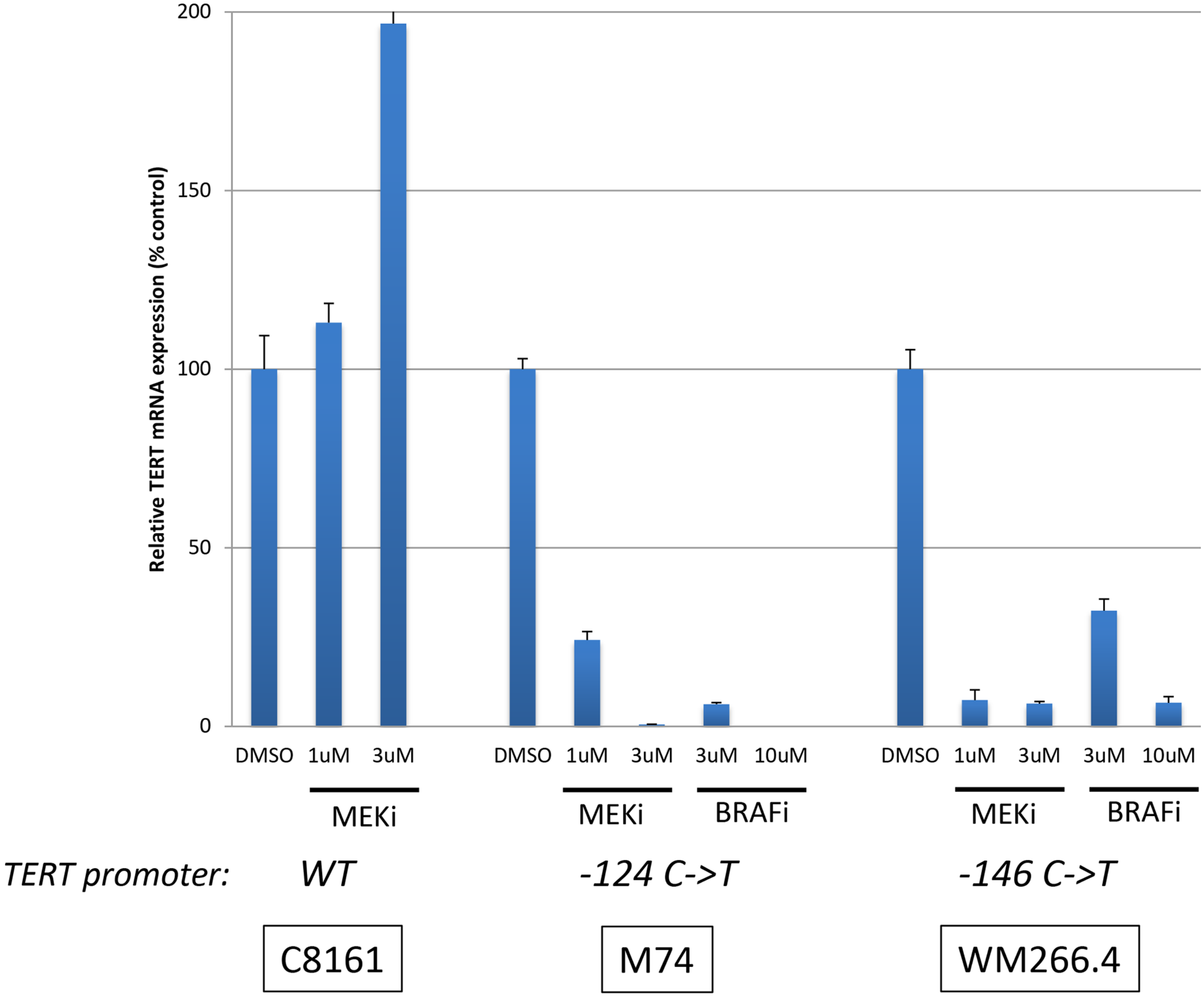

Supplementary Figure S1: TERT expression is dependent on MAPK pathway activation. Melanoma cell lines were treated for 24 hrs with 1 µM, 3 µM of Trametinib (MEKi), 3 µM, 10 µM of vemurafenib (BRAFi) or DMSO. TERT mRNA levels were quantified by real-time PCR normalized to GAPDH. Values are mean ± s.d. of two experiments assayed in duplicate. The TERT promoter status is indicated below the graph.

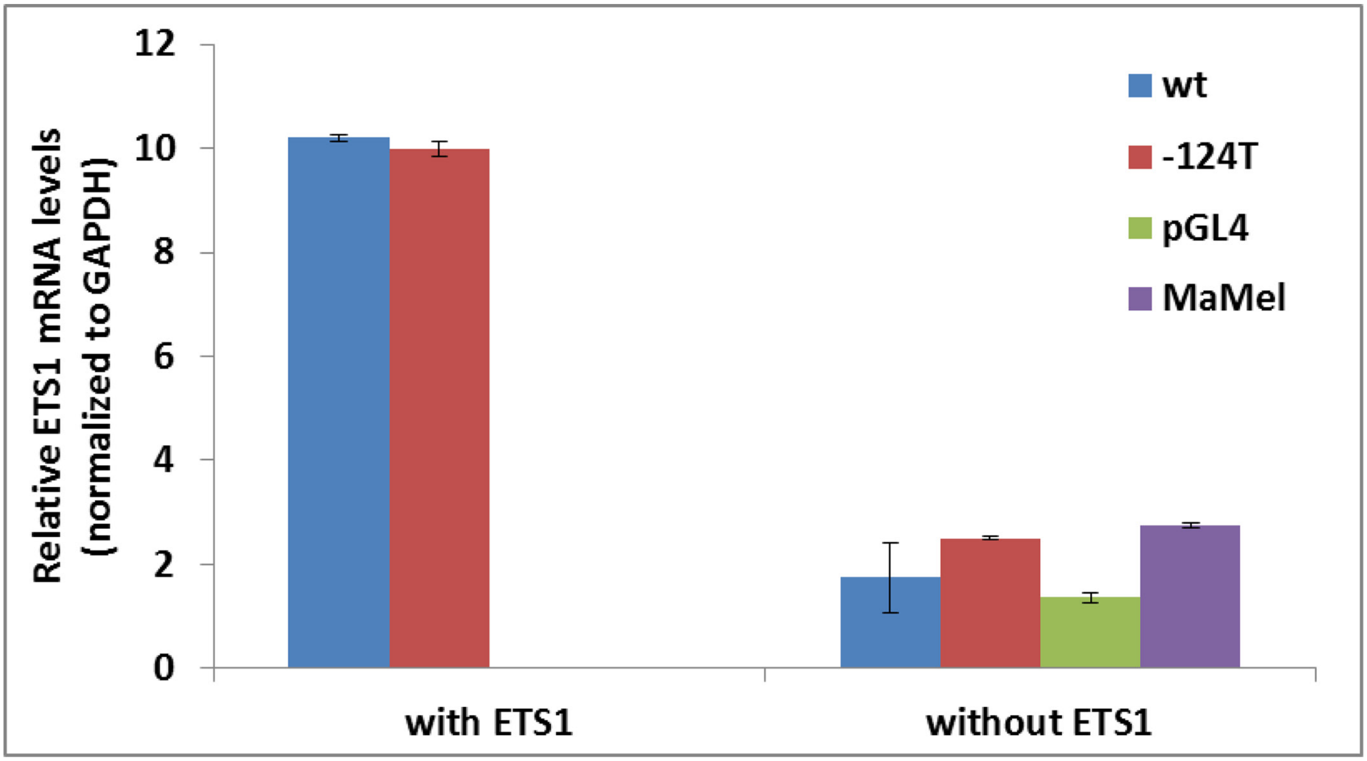

Supplementary Figure S2: Overexpression of ETS1 in cells transfected with ETS1 expression plasmid. Total RNA was extracted from the UKRV cells transfected with reporter constructs and with or without ETS1 expression plasmids. The ETS1 mRNA levels were quantified by real-time PCR normalized to GAPDH expression levels.

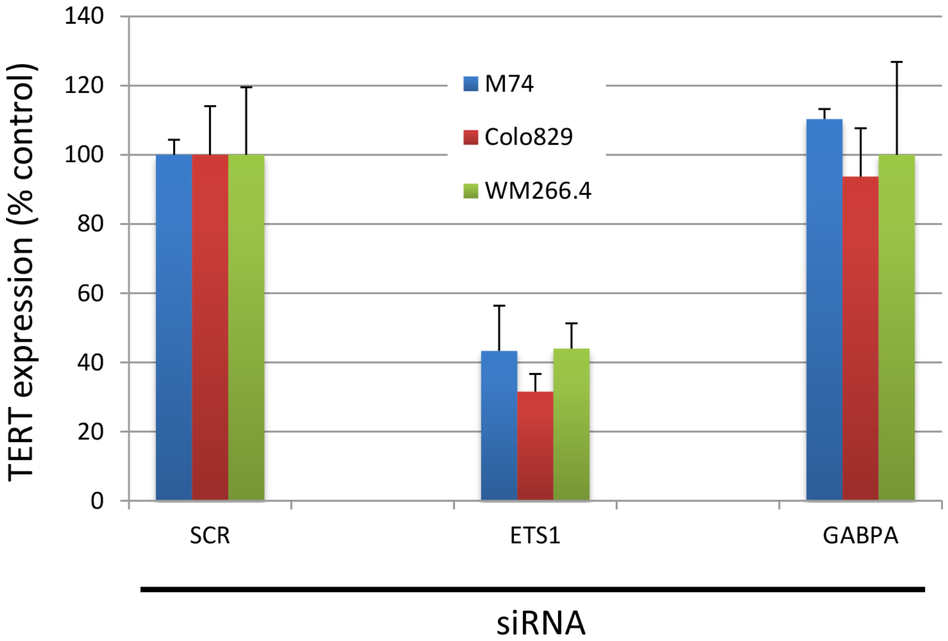

Supplementary Figure S3: Quantification of TERT expression in siRNA experiments presented Figure 3B. Melanoma cell lines were transfected without siRNA control (scr), siRNA targeting ETS1 or GAPBA. After 72 hrs, levels of TERT, ETS1 and GAPBA were analyzed by Western Blotting (Figure 3B). The level of TERT expression was quantified; data are represented as mean +/− SD.

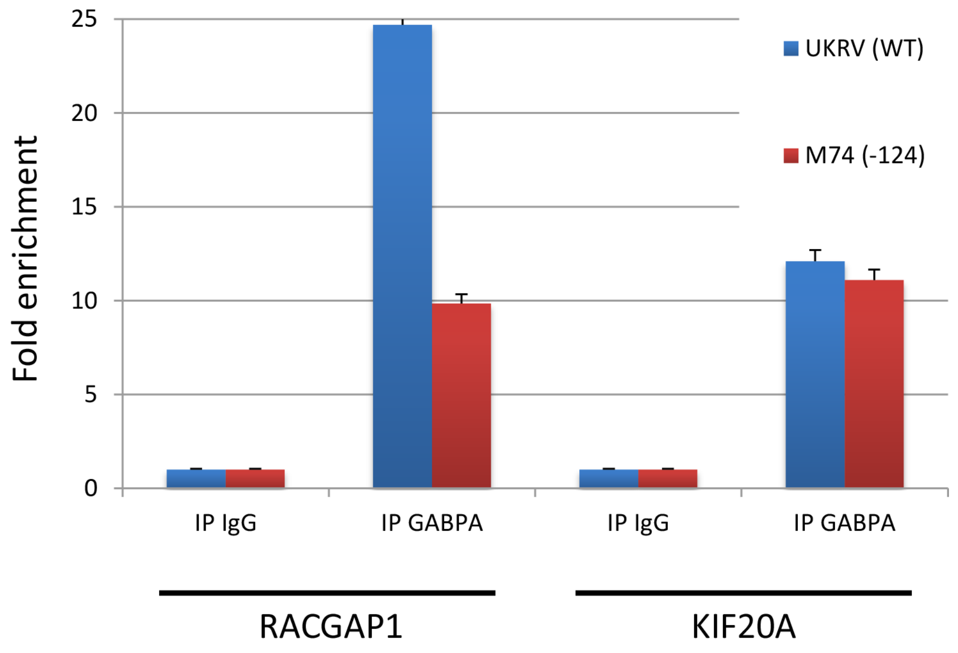

Supplementary Figure S4: GABPA binds to RACGAP1 and KIF20A promoters. Melanoma cell lines were formalin fixed and harvested. Chromatin precipitated by anti-GAPBA or control IgG was reverse-cross-linked, and the obtained genomic fragments were quantified by real-time PCR. Values are presented as fold enrichment over the mean value of control IgG. Error bars represent standard deviations.

Supplementary Table S1: Sequence of the siRNA and primers for QPCR and CHIP

Target	Séquence
siRNA-ets1a	GAAAGCAUAGAGAGCUACGAUAGUU
siRNA-ets1a	AACUAUCGUAGCUCUCUAUGCUUU
siRNA-ets1b	GGAGAUGGCUGGGAAUUCAAACUUU
siRNA-ets1b	AAAGUUUGAAUUCCCAGCCAUCUC
siRNA-gabpa1	GCUCUGGCUACUGCUUCUCUGCAAA
siRNA-gabpa1	AAAGCAGAGAAGCAGUAGCCAGAGC
siRNA-gabpa2	GGAGCUGAUAGAAAUUGAGAUUGAU
siRNA-gabpa2	AUCAAUCUCAAUUUCUAUCAGCUCC
QPCR-TERT-F	CATTTTTCCTGCGCGTCAT
QPCR-TERT-R	GCGACATCCCTGCGTTCT
QPCR-GADPH-F	TGGGTGTGAACCATGAGAAGTATG
QPCR-GADPH-R	GGTGCAGGAGGCATTGCT
CHIP-TERT	CTGCCCCTTCACCTTCCAG
CHIP-TERT	CTGCCTGAAACTCGCGCC
